# BMC Obesity reviewer acknowledgement 2015

**DOI:** 10.1186/s40608-016-0089-1

**Published:** 2016-02-10

**Authors:** Clare Partridge

**Affiliations:** BioMed Central, Floor 6, 236 Gray’s Inn Road, London, WC1X 8HB UK

## Abstract

The editors of BMC Obesity would like to thank all our reviewers who have contributed to the journal in Volume 2 (2015).

**Gavin Abbott**

Australia

**Asnawi Abdullah**

Indonesia

**Jesmin Akter**

Bangladesh

**AMAAP Alagiyawanna**

Sri Lanka

**Navoda Atapattu**

Sri Lanka

**Tom Barber**

UK

**Fiona Kate Barlow**

Australia

**Monica Baskin**

USA

**Sanjay Basu**

USA

**Grzegorz Bazylak**

Poland

**Alison Beauchamp**

Australia

**Rebecca Beeken**

UK

**Britni Belcher**

USA

**Colin Bell**

Australia

**Ragnar Bjarnason**

Iceland

**Catriona Bonfiglioli**

Australia

**Paula Brauer**

Canada

**Andrew Breck**

USA

**Anna-Kristin Brettschneider**

Germany

**Adrian Brown**

UK

**Maria Bryant**

UK

**Robert Caesar**

Sweden

**Adrian Cameron**

Australia

**Dexter Canoy**

UK

**Gianluca Castelnuovo**

Italy

**Mu Chen**

USA

**Mansi Chopra**

India

**Paul Collings**

UK

**Amanda Daley**

UK

**Hélène Delisle**

Canada

**Jacopo Desiderio**

Italy

**Hossam Draz**

Saudi Arabia

**John Dube**

USA

**Kate Ellacott**

UK

**Wayne English**

USA

**Eshan Fernando**

Canada

**Rebecca Firestone**

USA

**Helena Fonseca**

Portugal

**Rosanne Freak-Poli**

Australia

**L M Funk**

USA

**Frances Garden**

Australia

**Jewel Gausman**

USA

**Gilles Gouspillou**

Canada

**Josephine Gwynn**

Australia

**Nyssa Hadgraft**

Australia

**Mark Harris**

Australia

**Hongbo He**

China

**David Heber**

USA

**Peter Henneman**

Netherlands

**Erica Hinckson**

New Zealand

**Bruce Hollis**

USA

**Katy Horner**

Ireland

**Golam Hossain**

Bangladesh

**Haijing Huang**

USA

**Fumiaki Imamura**

UK

**Lindsay Jaacks**

USA

**Chandra Jackson**

USA

**G S Jakobsen**

Norway

**Randi Jepsen**

Denmark

**Rodney Joseph**

USA

**Nishan Kalupahana**

Sri Lanka

**Anne Kavanagh**

Australia

**Isurujith Kongalaliyanage**

Sri Lanka

**Vivica Kraak**

USA

**Yan Lam**

Australia

**Donatella Lanari**

Italy

**Rachel Laws**

Australia

**Dayna Lee-Baggley**

Canada

**Jimmy Chun Yu Louie**

Australia

**Julie Lumeng**

USA

**Brigid Lynch**

Australia

**Claire Madigan**

Australia

**Kristen Malecki**

USA

**Lucia Martinez De La Escalera Clapp**

USA

**Paolo Marzullo**

Italy

**Stephanie Mazzucca**

USA

**Philip McTernan**

UK

**Alexander Miras**

UK

**Jason Nagata**

USA

**Christine Olson**

USA

**Rocco Paluch**

USA

**Dimitris Papamargaritis**

UK

**Guillaume Pare**

Canada

**Ashan Pathirana**

Sri Lanka

**Christine Peat**

USA

**Melanie Pescud**

Australia

**Helena Piccinini-Vallis**

Canada

**Milan Piya**

UK

**Virginia Quick**

USA

**Elizabeth Reifsnider**

USA

**Jinnie Rhee**

USA

**Wendy Robertson**

UK

**Liane Roe**

USA

**Bashiru Imoro Ibn Saeed**

Ghana

**Francesca Seta**

USA

**Florian Seyfried**

Germany

**Carrie Sheets**

USA

**Saad Siddiqui**

Oman

**Jamie Sims**

UK

**Debbie Smith**

UK

**Nicola Spurrier**

Australia

**Claire Stocker**

UK

**Claudia Strugnell**

Australia

**Nicole Stupka**

Australia

**Aaron Sussell**

USA

**Abd Tahrani**

UK

**Lisa Te Morenga**

New Zealand

**Simon Thornley**

New Zealand

**Qingchun Tong**

USA

**Léonie Uijtdewilligen**

Singapore

**Cherian Varghese**

Switzerland

**Madhusudhan Varma**

UK

**Anthony Viera**

USA

**Ramon Vilallonga**

Spain

**Tommy Visscher**

Netherlands

**Indu Waidyatilaka**

Sri Lanka

**Connie Weaver**

USA

**Brandy Wicklow**

Canada

**Pujitha Wickramasinghe**

Sri Lanka

**Cameron Willis**

Canada

**Tsedeke Wolde**

Ethiopia

**Evelyn Wong**

Australia

**Yao Yao**

USA

**Alfred Yawson**

Ghana

**Changzheng Yuan**

USA

**Michael Zulyniak**

Canada

